# ^18^F-FDG-PET/CT as part of the diagnostic workup of native valve endocarditis: A case report

**DOI:** 10.1007/s12350-021-02849-7

**Published:** 2021-11-17

**Authors:** Christina Corby-Zauner, Pierre Monney, Matthaios Papadimitriou-Olivgeris, John O. Prior, Christel H. Kamani

**Affiliations:** 1 Department of Anesthesia, Lausanne University Hospital CHUV, Lausanne, Switzerland; 2 Department of Cardiology, Lausanne University Hospital CHUV, Lausanne, Switzerland; 3 University of Lausanne, Lausanne, Switzerland; 4 Department of Infectious Disease, Lausanne University Hospital CHUV, Lausanne, Switzerland; 5 Department of Nuclear Medicine and Molecular Imaging, Lausanne University Hospital CHUV, Lausanne, Switzerland

## Introduction

We illustrate the potential of ^18^F-fluodeoxyglucose (^18^F-FDG) Positron emission tomography (PET)/computed tomography(CT) in the detection of submitral native valve endocarditis (NVE).

## Case Presentation

**Figure 1:**
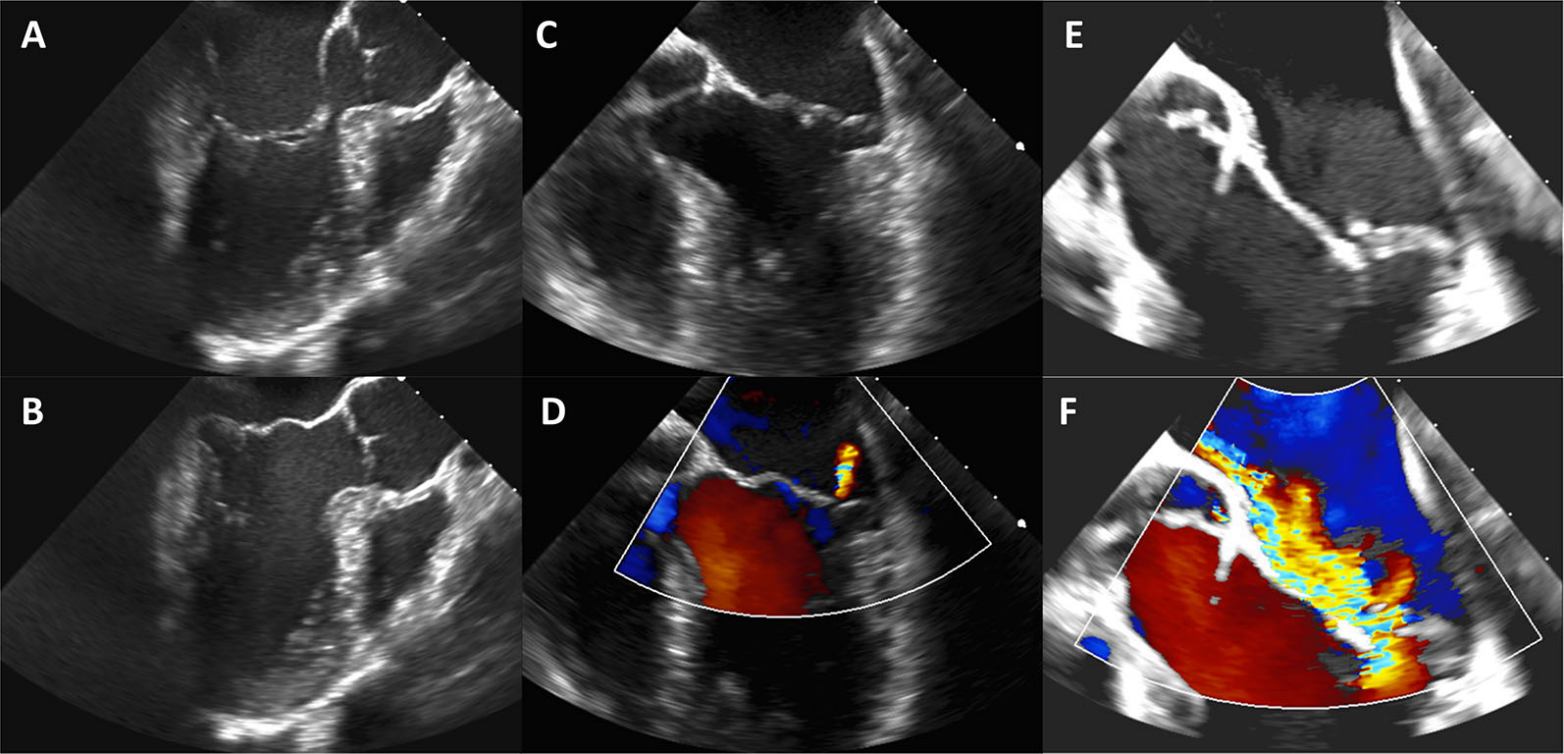
Transoesophageal echocardiographic findings. **A**, **B** Long-axis view in transesophageal echocardiogram (TOE) before antibiotic treatment in diastole (**A**) and systole (**B**) showing a billowing of the mitral valve, a finding already seen in a transthoracic echocardiocardiography (TTE) performed 3 days earlier. There were no evidence of vegetation or perivalvular abscess of the mitral valve. Moreover, no evidence of endocarditis of the submitral apparatus were seen in TTE as well as TOE. All other valves were normal. **C**, **D** Long-axis view in TOE (**C**) and the corresponding Color Flow Doppler (**D**) showing a mild mitral regurgitation. **E**, **F** Long-axis view in TOE (**E**) and the corresponding Color Flow Doppler (**F**) 6 weeks after completion of the antibiotic treatment demonstrating a severe eccentric mitral valve regurgitation with P2 prolaps secundary to chordea rupture. No vegetation or abscess were detected. Hemocultures were sterile and laboratory tests did not reveal any elevated inflammatory parameters (WBC 0.8 G/L; CRP < 1 mg/L). It is recognized that identification of vegetation using echocardiography may be difficult after recent embolization as it was the case in this patient. Since baseline TOE demonstrated only mild billowing of the posterior mitral leaflet, a spontaneous degenerative chordal rupture in a young patient would appear very unlikely in this situation, supporting our hypothesis that an infectious process limited to the sub-valvular apparatus led to the chordal rupture. Moreover, surgical inspection confirmed the isolated chordal rupture without damage of the mitral valve leaflet, also correlating with previous ^18^F-FDG PET/CT finding

**Figure 2:**
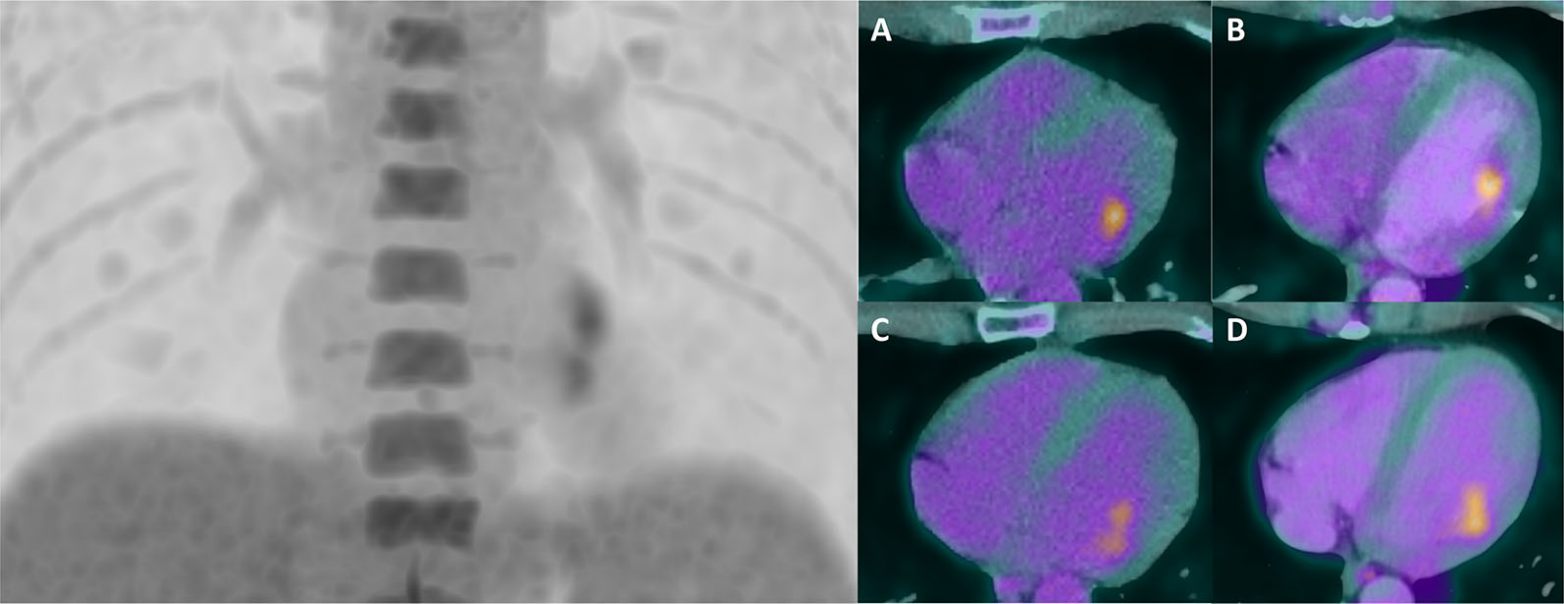
PET/CT and computed tomography angiography (CTA) findings. To assess for presence of extracardiac septic foci, a ^18^F-FDG PET/CT, performed after 3 days of dietary preparation and heparin pre-administration to suppress physiological myocardial FDG uptake was performed. **Left**, Maximum intensity projection image of the thorax showing pathological accumulation of ^18^F-FDG in the heart area. **Right**, **A**, **B** Transaxial view of ^18^F-FDG PET/CT (**A**) and ^18^F-FDG PET/CTA (**B**) showing focal FDG uptake of the chordea tendinae at the insertion in the anterolateral papillary muscle. **C**, **D** Transaxial view of ^18^F-FDG PET/CT (**C**) and ^18^F-FDG PET/CTA (**D**) showing focal FDG uptake of the chordea tendinae between the valve leaflet and the posteromedial papillary muscle. Due to the poor sensitivity, a negative ^18^F-FDG PET/CT cannot rule-out NVE. Based on its excellent specificity, positive valvular- or peri-valvular FDG uptake could be used to rule-in NVE, since no physiological FDG-uptake is expected in this area.^1^ When using ^18^F-FDG PET/CT as major criterion for the NVE diagnosis, with state-of-the art dietary patient preparation, contemporary PET/CT devices (better spatial resolution) as well as delayed cardiac acquisitions (better contrast resolution), the sensitivity of ^18^F-FDG PET/CT in the NVE diagnosis is increased up to 80%, without altering the excellent specificity.^2^ In NVE, valve leaflets and peri-annular area are the most commonly sites of cardiac FDG-uptake. Isolated involvement of the submitral apparatus in absence of myocardial FDG uptake are very rare, evoking inflammatory cardiomyopathy (sarcoidosis), intraventricular thrombus, neoplasm or infective endocarditis^3^

A 36-year-old male presented with a 1-week history of intermittent binocular diplopia and confusion, associated with low-grade fever. Physical examination and ECG were normal, and the laboratory tests revealed discretely elevated inflammatory parameters. Four pairs of blood cultures returned positive for *Streptococcus mitis*. Echocardiography was performed (Figure 1, Panel A–D), followed by ^18^F-FDG PET/CT (Figure 2). According to the modified Duke criteria (1 major and 2 minor criteria), NVE was considered as possible and endocarditis treatment was started. A follow-up echocardiography was performed after treatment (Figure 1, Panel E, F).

## Discussion

Infectious endocarditis is a life-threatening disease, requiring prompt and accurate diagnosis. ^18^F-FDG PET/CT belongs to the major ESC criteria for the diagnosis of prosthetic valve endocarditis. However, its clinical use in NVE is limited due to its poor sensitivity, as demonstrated by a recent meta-analysis.^1^ This poor sensitivity relies on the avascular nature of the vegetation, the limited spatial and temporal resolution of PET/CT devices, incomplete dietary patient preparation leading to incomplete suppression of the physiological FDG uptake, as well as prolonged antibiotic treatment prior PET/CT.^1^ There are evidences on isolated papillary muscle endocarditis.^3^ However, we hereby firstly reported the link between previous focal FDG uptake of the submitral apparatus and the occurrence of chordal rupture in the clinical follow-up, which could not be detected in previous investigations (TTE, TOE and CTA). This correlation was confirmed by surgical inspection of the mitral valve, as well as baseline TOE findings. Despite its limitation in NVE, ^18^F-FDG PET/CT could be considered by high NVE suspicion, even if echocardiography or CTA are negative, since isolated endocarditis of the submitral apparatus, only seen in ^18^F-FDG PET/CT, is possible, even if rare.

## References

[1] Kamani CH, Allenbach G, Jreige M, Pavon AG, Meyer M, Testart N (2020). Diagnostic performance of (18)F-FDG PET/CT in native valve endocarditis: Systematic review and bivariate meta-analysis. Diagnostics (Basel).

[2] Abikhzer G, Martineau P, Gregoire J, Finnerty V, Harel F, Pelletier-Galarneau M (2020). [(18)F]FDG-PET CT for the evaluation of native valve endocarditis. J Nucl Cardiol.

[3] Fang N, Zeng L, Jin F, Lin S, Wang YL (2021). 18F-FDG PET/CT in infective endocarditis on papillary muscles: A case report. Clin Nucl Med.

